# Photothermal Therapy *via* NIR II Light Irradiation Enhances DNA Damage and Endoplasmic Reticulum Stress for Efficient Chemotherapy

**DOI:** 10.3389/fphar.2021.670207

**Published:** 2021-04-29

**Authors:** Qingduo Kong, Dengshuai Wei, Peng Xie, Bin Wang, Kunyi Yu, Xiang Kang, Yongjun Wang

**Affiliations:** ^1^ Department of Obstetrics and Gynecology, Peking University International Hospital, Beijing, China; ^2^ Clinical Medical College, Weifang Medical University, Weifang, China; ^3^ Beijing National Laboratory for Molecular Sciences, State Key Laboratory of Polymer Physics and Chemistry, Institute of Chemistry, Chinese Academy of Sciences, Beijing, China; ^4^ Department of Obstetrics and Gynecology, Union Hospital, Tongji Medical College, Huazhong University of Science and Technology, Wuhan, China

**Keywords:** chemotherapy, photothermal therapy, mild hyperthermia, NIR II light, DNA damage, endoplasmic reticulum stress key

## Abstract

Ovarian cancer has the highest death rate in gynecologic tumors and the main therapy for patients with advanced is chemotherapy based on cisplatin. Additionally, hyperthermic intraperitoneal has been used in clinic to obtain better efficacy for patients. Hence, combined photothermal therapy with platinum drugs in a new delivery system might bring new hope for ovarian cancer. A reduction sensitive polymer encapsulating a Pt (IV) prodrug and a near infrared II (NIR II) photothermal agent IR1048 to form nanoparticles were reported to enhance the efficacy of ovarian cancer treatment. At the same time, endoplasmic reticulum stress indicates an imbalance in proteostasis which probably caused by extrinsic stress such as chemotherapy and the temperature changed. The efficacy of nanoparticles containing Pt (IV) and IR1048 under NIR II light might be caused *via* increased DNA damage and endoplasmic reticulum (ER) stress.

## Introduction

Ovarian cancer is one of the most common malignant tumors in gynecology. Once diagnosed, 70% of ovarian cancer patients are in advanced stage, and nearly no targeted drugs are available (Wright et al., 2016; Grossman et al., 2018). The main treatment of ovarian cancer is still chemotherapy based on cisplatin (Konner et al., 2011). However, cisplatin resistance as well as its serious side effects hindered its application (Rottenberg et al., 2021). To this end, the development of platinum drugs with high efficiency and low toxicity combined with a new delivery system might bring new hope (Wang et al., 2019; Xiaoxu et al., 2019).

Extensive progress has been made on photothermal therapy (PTT) recently (Yang et al., 2020; Wen et al., 2021). The mechanism of PTT is to convert light energy into local heat energy through photothermal agents, which can be used to eradicate tumor through thermal ablation (Meng et al., 2018). The advantages such as non-invasive operation, high specificity, make it a promising candidate for cancer therapy (Liu et al., 2011; Ruan et al., 2019; Jędrzak et al., 2020). Numerous PTT agents with high photo conversion efficiency (PCE) have been reported. However, the temperature over 50 C might lead to side effect such as skin burns. Hence, in comparison to conventional PTT which focuses on the high operation temperature, mild hyperthermia lower than 45°C is more desirable for clinical application. Furthermore, PTT with excitation light in NIR II window (1000–1700 nm) has drawn tremendous attention due to the reduced absorption and tissue scattering. It is able to penetrate deep tumor tissue, which is more suitable for the clinic practice (Chen et al., 2020; Yang et al., 2021). In practical clinical scenario, hyperthermic intraperitoneal has already been included in the guideline of 2019 NCCN Guidelines Insights for ovarian cancer (Armstrong et al., 2019).

The ability of cancer cells to respond to extrinsic stress such as chemotherapy or mild hyperthermia depends on their ability to activate appropriate adaptive pathways. In the endoplasmic reticulum (ER), ER stress indicates an imbalance in proteostasis which probably caused by extrinsic stress (Rubio-Patiño et al., 2018). Studies showed that ER stress might be a pathway which caused cell apoptosis (Madden et al., 2019). X-Box binding protein 1 (XBP1), which is an important protein in ER stress pathway, was significantly reduced under this combinational therapy (Chen et al., 2016; Lhomond et al., 2018).

Herein, we hypothesize that mild hyperthermia relying on NIR II laser combined with cisplatin chemotherapy could become a promising strategy to tackle with ovarian cancer (Balakrishnan et al., 2020; Xi et al., 2020). To achieve this, a reduction sensitive block polymer (P1) was firstly synthesized, which is characterized by the presence of numerous disulfide bonds (S-S) in the polymer main chain. Secondly, a hydrophobic Pt (IV) prodrug with aliphatic chains was prepared. Subsequently, P1 and Pt (IV) prodrug were mixed to self-assemble into nanoparticles with Pt (IV) prodrugs (NP-1). To obtain the final nanoparticles with photothermal effect, P1 and Pt (IV) prodrug as well as a IR1048 were mixed to self-assemble into nanoparticles with both Pt (IV) prodrugs and photothermal dyes (NP-2). After NP-2 enter cancer cells *via* endocytosis, they could then be dissociated to release toxic cisplatin in the reductive microenvironment, leading to DNA replication and transcription dysfunction (Mortensen et al., 2020; Zhang et al., 2020). At the same time, the released photothermal IR1048 can be irradiated under 1064 nm laser, resulting in photothermal effect which could further sensitize cisplatin *via* enhanced DNA damage and ER stress. We found here the combination of mild hyperthermia and cisplatin chemotherapy leads to decreased expression of X-Box binding protein 1 (XBP1), an important protein in ER stress pathway. Therefore, the combination of mild hyperthermia with platinum drugs in nanoparticles is of great significance and brings a new perspective to clinical treatment for ovarian cancer.

## Article Types

Original Research.

## Manuscript Formatting

### Materials and Methods

#### Materials

Cisplatin were purchased from Shangdong Boyuan Chemical Company. 3- (4,5-dimethyl-2-thiazolyl)-2,5-diphenyl-2-H-tetrazolium bromide (MTT), 2- (4-amidinophenyl)-1H-indole-6-carboxamidine (DAPI), methyl sulfoxide (DMSO), ethanol, sodium azide (NaN3), IR1048 were purchased from Sigma-Aldrich (Shanghai, China). RPMI 1640, fetal bovine serum (FBS), penicillin-streptomycin solution, trypsin, Annexin V-FITC/PI apoptosis detection kit and cell cycle detection kit were purchased from Keygen Biotech. Hydrogen peroxide, N, N-Dimethyl formamide (DMF) and methoxyl-poly (ethylene glycol) (molecular weight = 5000) were purchased from Beijing chemical works.

#### Preparation of Pt(IV)-OH

Cisplatin (1.67 mmol, 0.5 g) was suspended in H_2_O_2_. The mixture was stirred at 40 C overnight and clear solution was produced. After the mixture down to the room temperature, much of needle-like crystal was precipitated. The product was washed many times with acetone and dried in vacuum oven. The yield rate was 80%. ESI-MS (positive mode) for Cl_2_H_8_N_2_O_2_Pt: *m/z* [M + H]^+^ Calcd: 332.96, Found: 333.0.

#### Preparation of C_8_-Pt(IV)-C_8_ [Pt(IV)]

Pt (IV)-OH (1.2 mmol, 400 mg) and 1-isocyanatooctane (2.4 mmol, 0.744 ml) were suspended in 5 ml anhydrous DMF. The solution is stirred at 70 C for 8 h. The solvent is evaporated, the residual substance is dissolved by a small amount of methanol, and the methanol solution is added with ice ethyl ether under stirring to collect the precipitate products. Products were dried under the vacuum to obtain C8-Pt (IV)-C8 [Pt (IV)] (28%) as light yellow solid. ^1^H NMR (300 MHz, DMSO) 6.61 (7 H, d, J 43.6), 2.88 (2 H, d, J 6.0), 1.29 (12 H, d, J 31.9), 0.86 (3 H, t, J 6.5). ESI-MS (positive mode) for C_18_H_43_C_l2_N_4_O_4_Pt^+^: *m/z* [M + H]^+^ Calcd: 644.23, Found: 644.24.

#### Preparation of P1

DSB ((2,2′-disulfanediylbis (ethan-1-ol), 0.20 mmol, 30.8 mg) and CHTA (hexahydro-1H, 3H-benzo [1,2-c:4,5-c']difuran-1,3,5,7-tetraone, 0.21 mmol, 44.8 mg) were added in 5 ml anhydrous DMF. After magnetic stirring for 24 h, mPEG_5000_-OH (2 mmol, 110 mg) was added in the reaction system. After magnetic stirring for another 24 h, under sonication, 5 ml mixture was added in 15 ml deionized water, followed by dialysis in a dialysis bag (molecular weight cut-off, 5000 Da). After 72 h, the solution was freeze-dried under reduced pressure to obtained 112 mg polymer named P1.

#### Preparation of NP-1 and NP-2

20 mg P1 and 2 mg Pt (IV) were weighed and fully dissolved in 1 ml DMF. This mixture was stirred for 5 min. Then 10 ml water was added to the above solution in dropwise. After stirring for 10 min, it was subjected to dialysis for 12 h using dialysis bag (molecular weight cut-off, 3500 Da). The product was named NP-1. At the same time, 20 mg P1, 2 mg Pt (IV) and 1 mg IR1048 were weighed and fully dissolved in 1 ml DMF. This mixture was stirred for 5 min. Then 10 ml water was added to the above solution in dropwise. After stirring for 10 min, it was subjected to dialysis for 12 h using dialysis bag (molecular weight cut-off, 3500 Da). The product was named NP-2.

#### Drug Release of NP-2

Drug release of NP-2 was arisen at pH 7.4, pH 5.0 and in the presence of 10 mmol GSH, respectively. Different pH solutions were prepared in PBS. 1ml of nanoparticles solution was taken into dialysis bag (molecular weight cut-off, 3500 Da) and then immersed in those solutions of different pH as soon as possible. Then, the systems were placed into an incubator shaking at 37°C. At each specific point in time, 1 ml of sample solution was collected and replenish a considerable volume of solution immediately. All the samples were examined by ICP-MS.

#### Dissociation Kinetics of NP-2

The dissociation kinetics of NP-2 was performed according to report with minor modifications. In brief, P1 (10 mg) were dissolved in 1 ml DMF. 10 µl of Nile Red stock solution (0.05 mg/ml) in THF were added into the mixture. After that, with gentle stirring, 9 ml deionized water were added dropwise to prepare the new nanoparticle. The change in fluorescence curves were recorded at an excitation wavelength of 550 nm and the emission wavelength from 620 to 720 nm by adding 10 mM GSH at different time points. The half-life time (t_1/2_) was calculated based on the fluorescence measurements.

#### Hyperthermia Effect of NP-2

The hyperthermia effect of NP-2 was monitored under near-infrared light irradiation. Specifically, 1 ml of NP-2 at three different concentration (5.5, 11 and 22 μg/ml) was irradiated with 1064 nm near-infrared laser at three different powers (0.5, 1.0 and 1.5 W) respectively. The FLUKE thermal imager was used to determine the solution temperature and the temperature rise curve was recorded.

#### Cell Lines and Cell Incubation Conditions

A2780 and A2780 DDP cells were gifted from the Medical Department of Jilin University in China. A2780, A2780 DDP cells were incubated in RPMI 1640 media. Culture medium were supplemented with 10% fetal bovine serum, 1% penicillin-streptomycin solution. The cell lines were cultured in 37 C with 5% CO_2_ atmosphere.

#### Nanoparticles Uptake in the Cells

Fluorescent dye Rh B was encapsulated with the P1 to assemble new nanoparticles (termed NPs@Rh B). Cell slides were placed on the bottom of the wells in advance, then A2780 cells at a density of 2 × 10^4^ per well were seeded and incubated at 37°C overnight. The cells were exposed to NPs@Rh B with a final concentration of 2 μg/ml of Rh B for 1, 3 and 6 h. After that, cells were washed with cold PBS and fixed with paraformaldehyde. For cell colocalization, the nuclei were stained with DAPI (blue) and filamentous actin cytoskeleton were strained with phalloidin (green), respectively. Finally, images were immediately captured by confocal laser scanning microscopy (CLSM) at a × 100 magnification.

The intracellular uptake studies of NPs@Rh B were also performed by using flow cytometry. In particular, A2780 cells were seeded in 6-well plates with a density of 2 × 10^5^ cells per well. After incubation overnight, cells were exposed to the above synthesized NPs@Rh B for another 1, 3 and 6 h at 37 C. Next, cells were washed with PBS and harvested by trypsin, and the mean fluorescence intensity (MFI) of cells was detected by flow cytometry.

#### Cell Relative Viability Studies

MTT assay was used to examine the cell relative viability. A2780, A2780 DDP cells were seeded in 96-well plates with a density of 8 × 10^3^ per well and cultured at 37 C for 12 h. Cells were treated with cisplatin, NP-1, NP-2 and NP-2 with NIR II irradiation (after treatment for 18 h) with final concentrations (μM) of 0.25, 0.5, 1, 2, 4, 8 with Pt. After treatment for 24 h, 10% MTT diluted with 1640 was added in the wells. After incubation in 37 C for 4 h, 10% SDS was added in the wells and cells were incubated in 37 C for 12 h in the dark. The results were performed with Molecular Devices.

#### Apoptosis Studies

A2780 cells were seeded in twelve-well plates at a density of 2 × 10^5^ per well and incubated at 37 C for 12 h. Cisplatin, NP-1, NP-2, NP-2 with NIR II irradiation (after treatment for 6 h) were added in the wells at the final concentration 1 µm of Pt. After treatment for 12 h, the media was removed and the cells were washed Three times with cold PBS. Then the cells were harvested and strained with 5 µl FITC and 5 µl PI for 10 min in the dark at room temperature respectively. Finally, all the samples were performed with flow cytometry in 1 h.

#### Cell Cycle Studies

A2780 cells were seeded in twelve-well plates at a density of 2 × 10^5^ per well and incubated at 37 C for 12 h. Cisplatin, NP-1, NP-2, NP-2 with NIR II irradiation (after treatment for 6 h) were added in the wells at the final concentration of 1 µm of Pt. After treatment for 12 h, the media was removed and the cells were washed three times with cold PBS. After fixed with 50% ethanol at 4 C for 12 h, the cells were harvested and treated with RNAse (100 μg/ml) and propidium iodide (100 μg/ml) at 4 C for 30 min. Finally, all the samples were performed with flow cytometry in 1 h.

#### Western Blot Assay

In this study, the following primary antibodies were used: anti-PARP (# 9542, CST), XBP1 (# 40435, CST), HMGB1 (# 3935, CST) and GAPDH (# 5174, CST). Typically, A2780 cells were seeded in 6-well plates at a density of 6×10^5^ per well and incubated at 37 C for 12 h. Cisplatin, NP-1, NP-2, NP-2 with NIR II irradiation (after treatment for 18 h) at the final concentration of 1 µm of Pt for 24 h separately. Cells were harvested and the lysates were homogenized in RIPA lysis buffer followed by centrifugation (Beyotime, Beijing, China). A total of 30 μg protein was separated by SDS-PAGE and transferred onto a PVDF membrane. Following incubation with the primary antibodies overnight at 4 C, the membranes were washed with TBST and subsequently incubated with horse radish peroxidase (HRP)-conjugated anti-rabbit secondary antibody for 1 h. Bands of interest were analyzed by using image analysis system (Bio-rad, United States of America) according to the manufacturer's instructions. The lysates from the xenografts with different treatments were also homogenized in RIPA for western blot assay. GAPDH was used as the internal control.

#### Immunofluorescence of XBP1

Cell slides were placed on the bottom of the wells in advance, A2780 cells were seeded in 24-well plates at density of 1 × 10^5^ per well and incubated at 37 C for 12 h. Cisplatin, NP-1, NP-2, NP-2 with NIR II irradiation (after treatment for 18 h) at the final concentration of 1 µm of Pt were added. After treatment of 24 h, absorb liquid, and cover cells to a depth of 2–3 mm with 4% formaldehyde diluted in PBS. Cells were fixed for 15 min at room temperature. Then washed three times in PBS for 5 min each. Cells were blocked in blocking buffer for 60 min. Then they were applied diluted primary antibody. They were incubated overnight at 4 C. They were washed three times in PBS for 5 min each. And they were incubated specimen in fluorochrome-conjugated secondary antibody diluted in antibody dilution buffer for 2 h at room temperature in the dark. They were washed three times in PBS for 5 min each. Coverslip slides with DAPI. In the end, images were performed with CLSM.

#### Flow Cytometry of XBP1

A2780 cells were seeded in 6-well plates with a density of 2 × 10^5^ cells per well. After incubation overnight, incubated with Cisplatin, NP-1, NP-2, NP-2 with NIR II irradiation (after treatment for 18 h) at the final concentration of 1 µm of Pt. After treatment, 1 million cells in approximately were in 100 µl 4% formaldehyde. They were fixed for 15 min at room temperature. They were washed by centrifugation with excess PBS. Then, they were added 1 ml PBS and added ice-cold 100% methanol slowly to pre-chilled cells, while gently vortexed, to a final concentration of 90% methanol for 10 min on ice. And they were washed by centrifugation in excess PBS to remove methanol. They were added 100 µl of diluted primary antibody and incubated for 1 h at room temperature. They were washed by centrifugation in PBS for three times and added 100 µl of diluted fluorochrome-conjugated secondary antibody. And they were incubated for 30 min at room temperature with dark and washed by PBS for three times. They were added 500 µl PBS and analyzed on flow cytometer.

#### Statistical Analysis

Results are presented as mean ± standard deviation (S.D.). Statistical analysis is subjected to t testing and means are assessed for significance by using Student’s t-test. Differences are considered significant for *p* value less than 0.01 (*p < 0.01) and very significant for *p* value less than 0.01 (****p < 0.0001). Unless especially mentioned, all assays were conducted in triplicate in three independent experiments. All statistical analysis are performed by using SPSS.

### Results

#### Characterizations of NP-2

**FIGURE 1:**
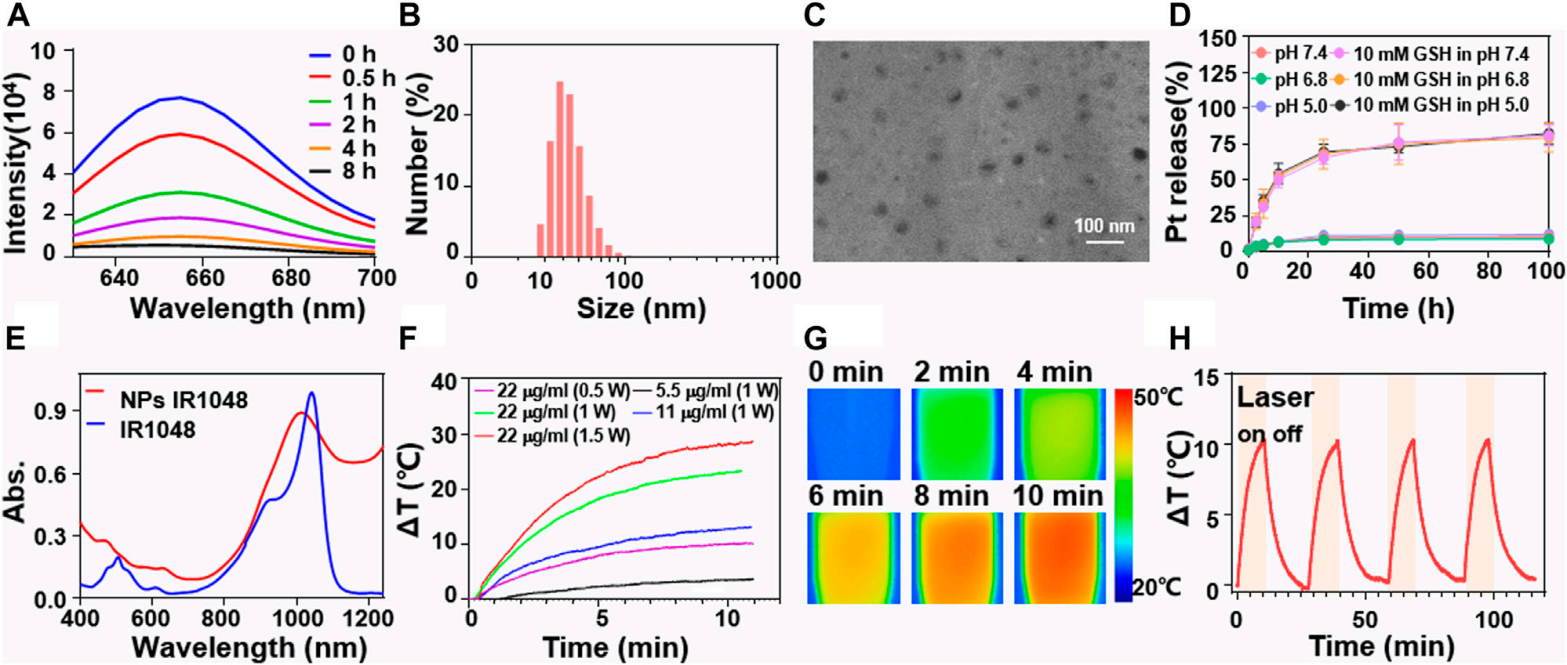
Characterizations of NP-2 with both Pt(IV) prodrugs and NIR II dye IR1048. The fluorescence curve at different time points after treatment of NP-2 with 10 mM GSH **(A)**. DLS of NP-2 **(B)**. TEM images of NP-2 **(C)**. Cumulative Pt release of NP-2 at pH 7.4, pH 5.0 and in 10 mM GSH aqueous solutions **(D)**. The UV−VIS-NIR spectra of IR1048 and NP-2 **(E)**. Photothermal curves of NP-2 under different conditions **(F)**. Thermo-graphic images of NP-2 (11 μg/ml) under 1064 nm laser irradiation (1 W/cm^2^) for 10 min **(G)**. The temperature variation profiles of NP-2 (11 μg/ml) in four successive cycles of laser on (10 min, 1 W/cm^2^) and off processes under 1064 nm laser irradiation **(H)**.

The combination of mild hyperthermia with platinum drugs in nanoparticles is of great significance and brings a new perspective to clinical treatment for ovarian cancer. P1 was successfully synthesized and characterized. Its molecular weight is about 15000 Da (Supplementary Figure S1). The Pt (IV) prodrug with axial octyl groups was then characterized by 1HNMR (Supplementary Figure S2). To confirm the responsiveness of NP-1 and NP-2 to GSH, Nile red dye was encapsulated. The results showed that the fluorescence intensity of the polymer containing Nile red gradually decreased with a half-life of 0.8 h (Figure 1A). Desirable NP-2 with both Pt (IV) prodrug and IR1048 were obtained at a feed ratio of Pt (IV), IR1048 and polymer was 2:1:20. Dynamic light scattering (DLS) showed that the size of NP-2 was 45 nm in diameter (Figure 1B). Transmission electron microscopy (TEM) showed that NP-2 were spherical with a mean diameter 40 nm (Figure 1C). Meanwhile, *in vitro* release profile of NP-2 showed that the cumulative platinum release was different at pH 7.4, pH 5.0 and in 10 mM GSH solutions. Specifically, the cumulative release was only 20% under pH 7.4 and pH 5.0, while the cumulative Pt release reached 80% in 10 mmol GSH (Figure 1D). The ultraviolet-visible-near-infrared (UV-VIS-NIR) absorption spectrum was further applied to study the optical properties and the potential for photothermal conversion. The UV-VIS-NIR absorption spectrum showed that NP-2 had an absorption peak at 1028 nm (Figure 1E). Thereafter, the photothermal effect of NP-2 with various concentration (5.5, 11 and 22 μg/ml) under different powers (0.5, 1.0and 1.5 W/cm^2^) was studied under the irradiation of 1064 nm laser. NP-2 can induce obvious photothermal effect, and the highest temperature increase (deta T) can reach 30°C (Figure 1F). Additionally, the temperature of NP-2 with the concentration of 11 μg/ml increased nearly 10°C which might be an appropriate temperature for mild hyperthermia under the stable light irradiation of 1064 nm for 10 min in Figure 1F. To visualize the increasing temperature, thermal imaging of NP-2 under the stable light irradiation of 1064 nm for 10 min were shown (Figure 1G). In addition, NP-2 had little photo-bleach effect during the four heating processes, indicating its excellent photothermal stability (Figure 1H).

#### Intracellular Uptake of NP-2 and Anticancer Activity Evaluation

**FIGURE 2:**
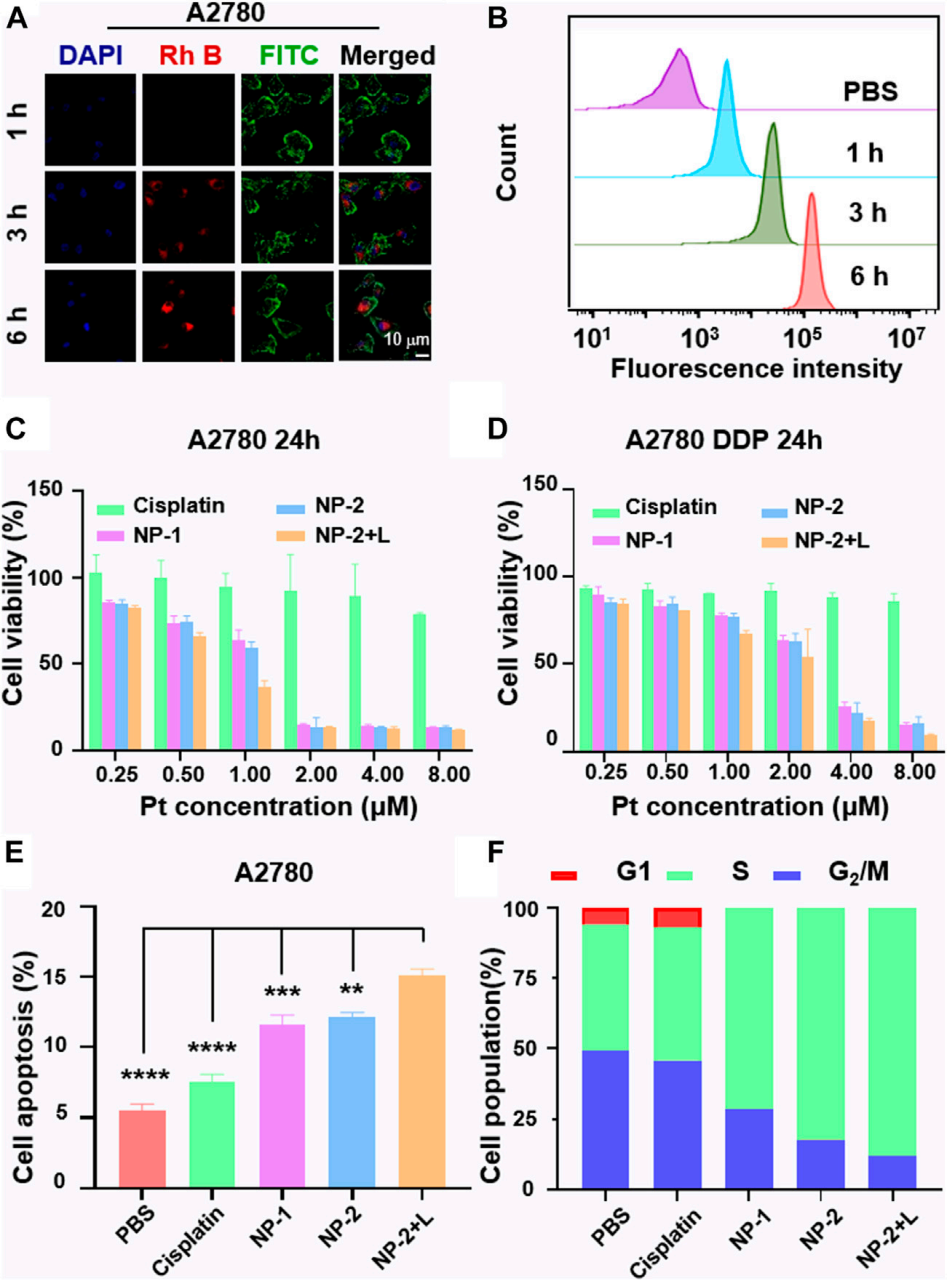
The intracellular uptake of NP-2 and the anticancer activity evaluation. CLSM images of A2780 cells incubated with nanoparticles labelled with Rh B for 1, 3 and 6 h respectively. The blue fluorescence comes from a nuclear dye DAPI. The red fluorescence comes from Rh B in the nanoparticles. The green fluorescence comes from a filamentous actin cytoskeleton dye FITC. The scale bar is 10 μm **(A)**. Quantification of the intracellular uptake of NP-2 on A2780 via flow cytometry **(B)**. The anticancer activity study of Cisplatin, NP-1, NP-2, NP-2 + L on A2780 and A2780 DDP cells via MTT assay **(C)** and **(D)**. Cell apoptosis rate induced by Cisplatin, NP-1, NP-2, NP-2 + L on A2780 cells **(E)**. Significance is defined as **p < 0.01, ***p < 0.001, ****p < 0.0001. Cell cycles of Cisplatin, NP-1, NP-2, NP-2 + L in A2780 cells **(F)**.

To visualize and quantify cell uptake of NP-2, P1 were labeled with rhodamine B (Rh B) (NPs@Rh B). NPs@Rh B was co-incubated with A2780 cells for 1, 3 and 6 h respectively. Subsequently, confocal laser scanning microscopy (CLSM) revealed that the red fluorescence in the cells increased with longer culture time (Figure 2A). In addition, flow cytometry quantitatively proved that the fluorescence intensity in A2780 cells increased with longer culture time which was nearly ten times at 6 h compared with PBS group indicating a rapid uptake of nanoparticles (Figure 2B and Supplementary Figure S3). Secondly, MTT assay was used to evaluate the cytotoxicity of cisplatin, NP-1 and NP-2 on A2780 and A2780 DDP cells *in vitro*. The results showed that NP-2 under NIR II light irradiation on A2780 cells and A2780 DDP cells were most potent (IC50 = 0.8 µM, IC50 = 2.2 µM). However, cisplatin was less potent on A2780 cells and A2780 DDP cells (IC50 >> 8 μM, IC50 >> 8 μM, Figures 2C,D). Similarly, NP-2 induced an apoptosis rate up to 14.61% on A2780 cells under the NIR II laser irradiation (Figure 2E and Supplementary Figure S4). Moreover, the S-phase arrest of cells treated with NP-2 under NIR II light was significantly higher than other treatment groups, suggesting that NP-2 may enhance DNA damage and induce more apoptosis (Figure 2F and Supplementary Figure S5).

#### DNA Damage and ER Stress in Cells Treated With NP-2 Under NIR II Light Irradiation

**FIGURE 3:**
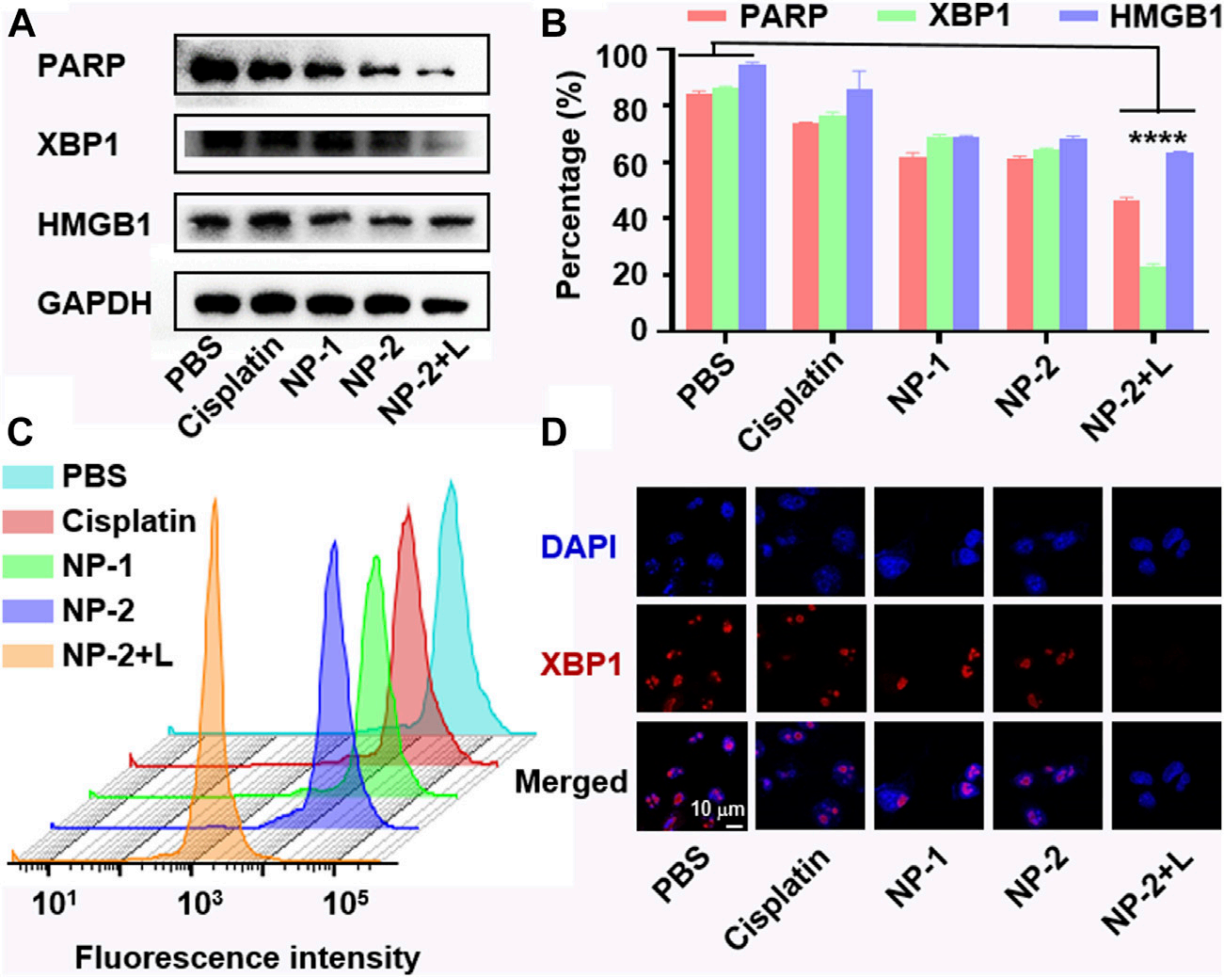
Increased DNA damage and ER stress in cells treated with NP-2 under NIR II light irradiation. Expression of PARP, XBP1, HMGB1 protein in cells treated with Cisplatin, NP-1, NP-2, NP-2+L **(A)**. Quantification of PARP, XBP1, and HMGB1 level in A **(B)**. Quantification of the XBP1 level in A2780 cells via flow cytometry **(C)**. CLSM images showing translocation of XBP1 in A2780 cells treated with Cisplatin, NP-1, NP-2, and NP-2+L respectively **(D)**. Significance is defined as ****p < 0.0001.

Platinum drugs inhibit cancer cell proliferation by increasing DNA damage (Yang et al., 2019). As a DNA damage repair enzyme, PARP is involved in the DNA damaging repair process (Shao et al., 2020). The expression of PARP might be closely related to apoptosis. As shown in Figures 3A,B, the expression of PARP in cells treated with NP-2 under NIR II irradiation significantly decreased, suggesting that NP-2 induced apoptosis by increasing DNA damage. At the same time, ER stress is a downstream mechanism of DNA damage which could promote cell apoptosis (Chen et al., 2018). As a biomarker of ER stress, XBP1 might be another reason for anticancer activity of NP-2 under 1064 nm laser irradiation. Western blot results further showed that the expression of XBP1 was significantly reduced in cells treated with NP-2 under NIR II light irradiation, suggesting an ER stress related pathway (Figures 3A,B).

High mobility group box one (HMGB1) is a nearly discovered inflammatory cytokine with immune stimulation. Studies showed that HMGB1 might induce cell apoptosis (Liu et al., 2021). Thereafter, we showed that HMGB1 levels in A2780 cells treated with NP-2 group under NIR II light irradiation decreased (Figures 3A,B). This suggested that mild hyperthermia by NIR II light might inhibited cell growth through immune-related responses. Moreover, flow cytometry analysis showed that cells treated with NP-2 under NIR II light irradiation had two times less XBP1 than other treatment groups (Figures 3C; Supplementary Figure S6). What’s more, CLSM showed that the XBP1 expression level of cells treated with NP-2 was very low under NIR II light irradiation, while the red signal of XBP1 expression was obvious in other treatment groups (Figure 3D).

### Conclusion

In conclusion, a reduction sensitive polymer was designed to encapsulate Pt (IV) prodrugs and NIR II photothermal dyes for nanoparticle-based drug delivery system. The nanoparticles can be dissociated in response to intracellular concentration of GSH, which could then effectively release cisplatin and IR1048. Moreover, the cancer cells can uptake the nanoparticles which were then heated under NIR II light irradiation, resulting in the enhanced DNA damage of platinum drugs, inducing ER stress, finally resulting in cell apoptosis, and possibly causing inflammatory and immune responses. Therefore, we found that mild hyperthermia under NIR II light irradiation could increase DNA damage and induce ER stress to enhance chemotherapy effect.

**SCHEME 1:**
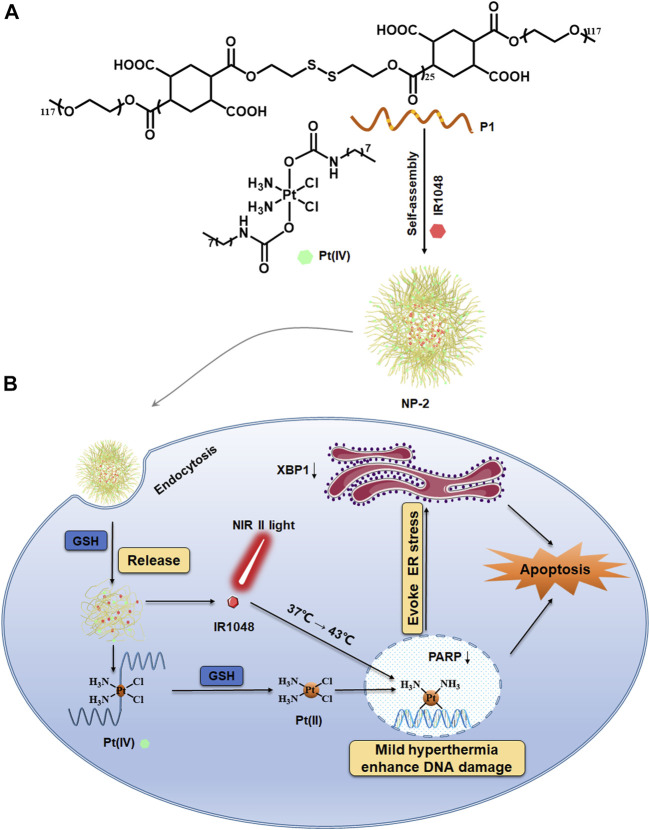
Mild hyperthermia induced by NIR II light irradiation enhancing platinum drug-based chemotherapy via increased DNA damage and ER stress. Chemical structure of the reductive polymer P1 and the Pt (IV) prodrug **(A)**. NP-2 were taken in by the cancer cells and the mild hyperthermia for enhancing chemotherapy via increased DNA damage as well as ER stress **(B)**.

## Data Availability

The original contributions presented in the study are included in the article/Supplementary Material, further inquiries can be directed to the corresponding authors.
