# Supplementary material for: Photothermal Therapy via NIR II Light Irradiation Enhances DNA Damage and Endoplasmic Reticulum Stress for Efficient Chemotherapy
Source: Front Pharmacol. 2021 Apr 29;12:670207. doi: 10.3389/fphar.2021.670207 (PMC8117088; doi:10.3389/fphar.2021.670207)
Supplement: Supplementary file 1 [file DataSheet1.docx]

Supplementary Material

**Table of Contents**

**Scheme S1 S2**

**Figure S1 S3**

**Scheme S2 S4**

**Figure S2 S5**

**Scheme S3 S6**

**Figure S3 S7**

**Figure S4 S8**

**Figure S5 S9**

**Figure S6 S10**

**Scheme S1. Synthesis route of Pt(IV).**

**
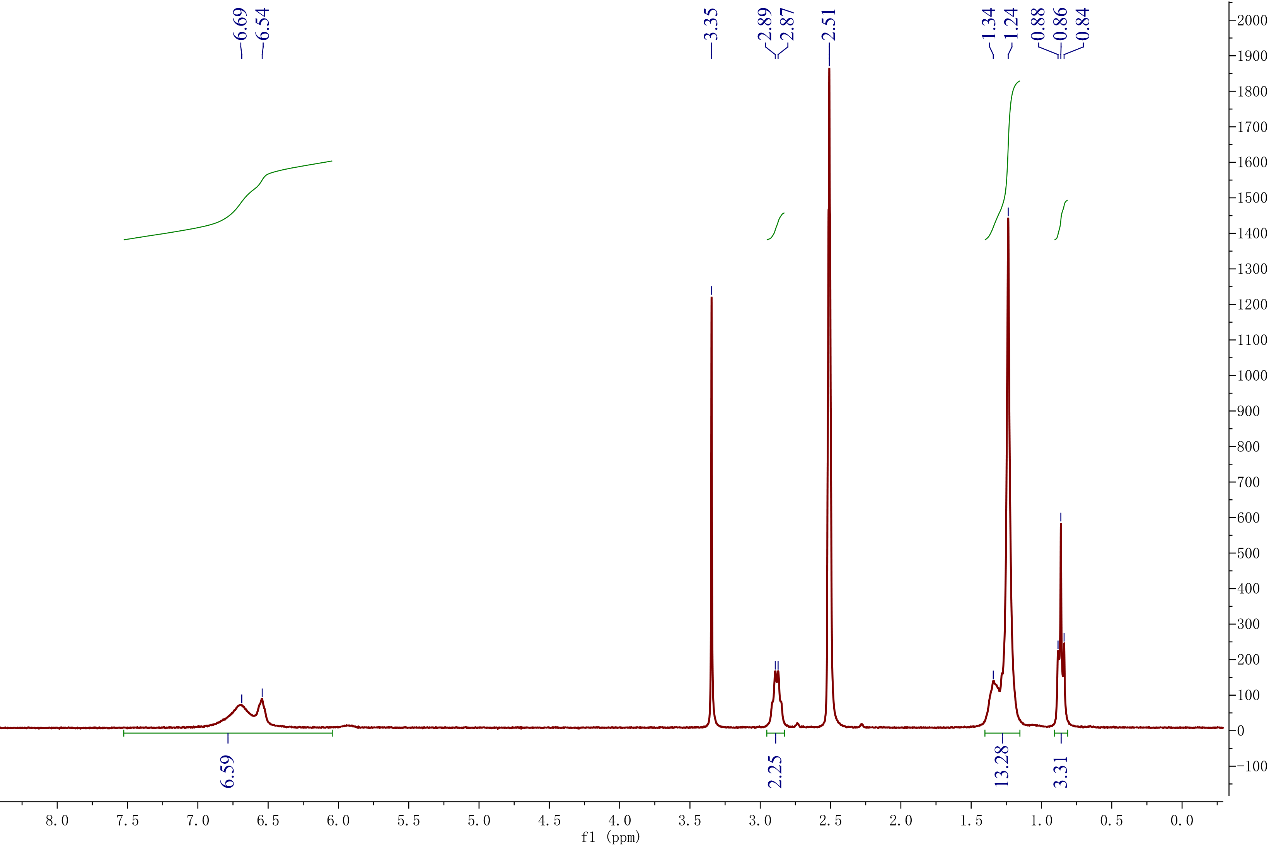
**

**Figure S1. Characterization of Pt(IV) by ^1^HNMR.**

**Scheme S2. Synthesis route of P1.**

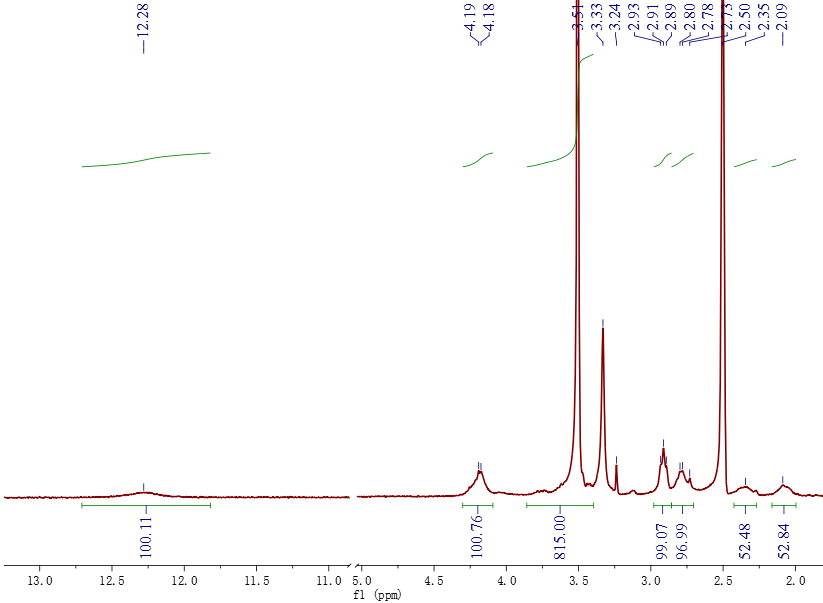


**Figure S2 ^1^H NMR Spectrum of P1.**

**Scheme S3. NP@Rh B formation as well as Rh B labelling.**

**Figure S3 Qualification of the NP@Rh B uptake on A2780 by flow cytometry**

**Figure S4 Apoptosis assay of cisplatin, NP-1, NP-2, NP-2+Light in A2780 cells.**

**Figure S5 Cell cycle study of A2780 cells after Cisplatin, NP-1, NP-2,NP-2+L treatment.**

**Figure S6 Quantification of the XBP1 in A2780 cells by flow cytometry**
